# HARVEST: A General-Purpose Platform for Mean-Field and Full-Field Composite Micromechanics and Its Validation with Polymer-Based Nanocomposites

**DOI:** 10.3390/polym18182213

**Published:** 2026-09-11

**Authors:** Mertol Tüfekci

**Affiliations:** 1 Centre for Engineering Research, College Lane Campus, University of Hertfordshire, Hatfield AL10 9AB, UK; m.tufekci@herts.ac.uk; 2 School of Physics, Engineering and Computer Science, College Lane Campus, University of Hertfordshire, Hatfield AL10 9AB, UK

**Keywords:** polymer-based nanocomposites, composite micromechanics, general-purpose simulation platform, software and physical-model validation, Mori–Tanaka homogenisation, representative volume element, interphase, periodic boundary conditions, CalculiX

## Abstract

Composite micromechanics is commonly divided between rapid mean-field estimates and computationally intensive full-field representative-volume-element (RVE) simulations. When these routes use different files, conventions and post-processing procedures, discrepancies can reflect bookkeeping rather than mechanics. This paper introduces HARVEST (Homogenisation and Representative Volume Element Simulation Tool; version 0.7.0.dev0), a general-purpose platform that coordinates mean-field homogenisation, three-dimensional RVE generation, finite-element model preparation, solver execution, homogenisation, parameter studies and post-processing through common project, service and provenance boundaries. The numerical framework is material-agnostic, whereas verification and validation are demonstrated using polymer-based nanocomposites. The Mori–Tanaka bulk response for spherical inclusions reproduces the Hashin composite-sphere result to machine precision, independent orientation procedures agree to a relative difference of $1.6\times 10^{-14}$, and a sequential coated-particle approximation differs from an analytical composite-sphere reference by at most 0.417% over 24 polymer-relevant configurations. An archived full-field epoxy/silica-type campaign using kinematic uniform boundary conditions and $20^{3}$ structured cells remains within the Hashin–Shtrikman interval at five inclusion fractions, with realisation scatter below 0.4%. For published epoxy nanocomposites, aligned halloysite-nanotube predictions differ from measured flexural moduli by −0.89 and +0.48%, while spherical carboxyl-terminated butadiene–acrylonitrile-rubber predictions differ by −7.03 and −2.25%. The main conclusion is that a shared, traceable description of constituents, morphology, loading and outputs supports rapid mean-field screening followed by selective full-field analysis using the same material definition. The principal advantage over single-route or loosely coupled workflows is cross-route consistency and reproducibility. HARVEST is applicable to formulation screening, sensitivity studies and local-field assessment in particulate, tubular, rubber-modified, porous and mixed-matrix polymer systems, and can be extended through validated constitutive, geometry, solver and result adapters.

## 1. Introduction

The performance of a polymer composite is governed by more than the nominal properties of its constituents. Filler volume fraction, aspect ratio, orientation, spatial arrangement, interphase behaviour and porosity all influence the effective stiffness and the local stress field. These effects are central to particulate thermosets, short-fibre thermoplastics, mixed-matrix membranes, rubber-toughened polymers and polymeric foams. Experimental characterisation remains indispensable, but a modelling route that connects constituent-scale descriptions to macroscopic response can reduce the number of candidate formulations that must be manufactured and can identify which microstructural variables deserve experimental attention.

Two modelling scales are used most often. Mean-field homogenisation, especially Eshelby-based Mori–Tanaka schemes [1,2,3,4], predicts effective properties from phase stiffnesses and compact morphological descriptors at negligible computational cost. Full-field homogenisation resolves an explicit RVE and local fields through a numerical boundary-value problem [5,6,7]. The latter can represent spatial interactions and local stress concentrations but introduces additional choices concerning RVE size [8,9,10], boundary conditions [11,12], discretisation and statistical sampling.

Tüfekci et al. [13] compared mean-field homogenisation with voxel- and tetrahedral-mesh FE models, examining the effects of inclusion aspect ratio and RVE size on accuracy and computational demand. For discontinuous-fibre composites, the two-dimensional calculations of Qian et al. [14] further relate the required representative size to fibre length and volume fraction. These studies motivate treating discretisation, morphology and statistical representativeness as separate modelling choices.

For polymer systems, these choices interact with unusually large modulus contrasts and with morphology-sensitive phenomena. Silica or halloysite can stiffen an epoxy matrix, whereas rubber particles and voids invert the contrast and reduce the elastic modulus. Nanotubes and nanofibrils require an orientation convention. Interphase layers may occupy a volume comparable with the nanofiller itself. Rate-dependent matrices additionally motivate viscoelastic or elastoplastic constituent models. Previous polymer studies have therefore combined experiments with mean-field and finite-element (FE) homogenisation for epoxy nanocomposites [15,16], polyacrylonitrile membranes [17,18], polyethersulfone membranes [19], and nanocellulose-reinforced membrane systems [20,21]. These studies also show that a single modelling route is not uniformly preferable across all morphologies.

For nanoscale reinforcements, length-scale effects and mechanical restraint introduce additional model choices. Tepe analysed the vibrations of single-walled carbon nanotubes on elastic foundations using nonlocal elasticity [22], and extended the approach to double-walled nanotubes with interwall coupling and foundation support [23]. Her nonlocal Timoshenko formulations examine nanobeam vibration with and without Winkler-foundation support [24,25]. A strain-gradient comparison of boron nitride and silicon carbide nanotubes considers material and higher-order boundary-condition effects [26], while Tepe and Güçlü [27] investigate material gradation in nonlocal nanobeam buckling. These studies address the response of individual nanotubes and nanobeams. They complement homogenisation by identifying constituent-scale effects that may require a richer description than a local phase stiffness alone.

A practical difficulty is that the two routes are frequently operated as unrelated workflows. Aspect ratio may be defined along a different local axis, mass and volume fractions may be interchanged, random and aligned orientation assumptions may be left unstated, and post-processing may report either a directional modulus or an isotropic projection. A numerical difference can then be caused by convention drift rather than by the underlying method. A second challenge is establishing independent evidence: agreement with another software package can be inconclusive when both implementations share the same assumptions. Software verification requires independent analytical identities, limiting cases and structural tests; physical validation requires separate comparison with experimental evidence.

Existing commercial and research tools cover different portions of this workflow. Digimat provides a broad multiscale environment for plastics, composites, metals and elastomers, including material, manufacturing and structural links [28]. Ansys Material Designer generates and analyses RVEs to obtain homogenised properties within the Workbench environment [29]. EasyPBC automates periodic constraints and elastic homogenisation for user-created Abaqus RVEs [11], while MicroStructPy concentrates on statistically controlled microstructure generation and meshing [30]. Table 1 compares their published scope with that of HARVEST. The comparison is functional rather than a runtime or accuracy ranking because a fair numerical benchmark would require common geometries, constitutive assumptions, solvers, meshes and hardware.

Complementary developments address particular stages of the analysis. Riaño and Joliff [31] provide geometry generation for randomly distributed unidirectional fibres and their interphases, while Ogierman [32] combines Mori–Tanaka and FE treatments of reinforcement shape and orientation. These contributions place integrated workflows within a wider set of specialised micromechanical methods.

The contribution of HARVEST is the traceable coordination of analytical mean-field and explicit full-field routes through common constituent definitions, morphology conventions, deterministic studies and result provenance. It links screening, microstructure generation, full-field solution and local-field interpretation within one research workflow, while mature commercial suites retain broader constitutive and process-modelling coverage.

HARVEST was developed to address these workflow and evidence gaps as a general composite micromechanics platform. The current development version provides a canonical command interface, typed project and run boundaries, mean-field and full-field services, solver management, parameter studies and post-processing. Established numerical modules remain available through compatibility adapters while the software architecture is migrated incrementally.

The contributions of the present paper are fivefold:

1. the current HARVEST architecture, implemented capabilities and scientific scope are documented;
2. the governing conventions used to connect mean-field and full-field analyses are stated explicitly, including the local-axis definition of spheroidal aspect ratio and the distinction among kinematic, static and periodic RVE conditions;
3. the mean-field and coated-particle kernels are tested against independent analytical references and limiting reductions;
4. a polymer-focused design study quantifies the effects of stiff, soft and void inclusions, halloysite aspect ratio and orientation, interphase stiffness and published epoxy/HNT and epoxy/CTBN measurements; and
5. all paper-specific calculations and figures are organised in a self-contained reproduction layer that explicitly separates recomputed evidence from archived aggregate FE data.

The study establishes what the current software calculates, how those calculations are traced, and which conclusions are supported for the polymer systems considered.

## 2. Software Architecture and Governing Formulations

### 2.1. Current Development Scope

**Figure 1:**
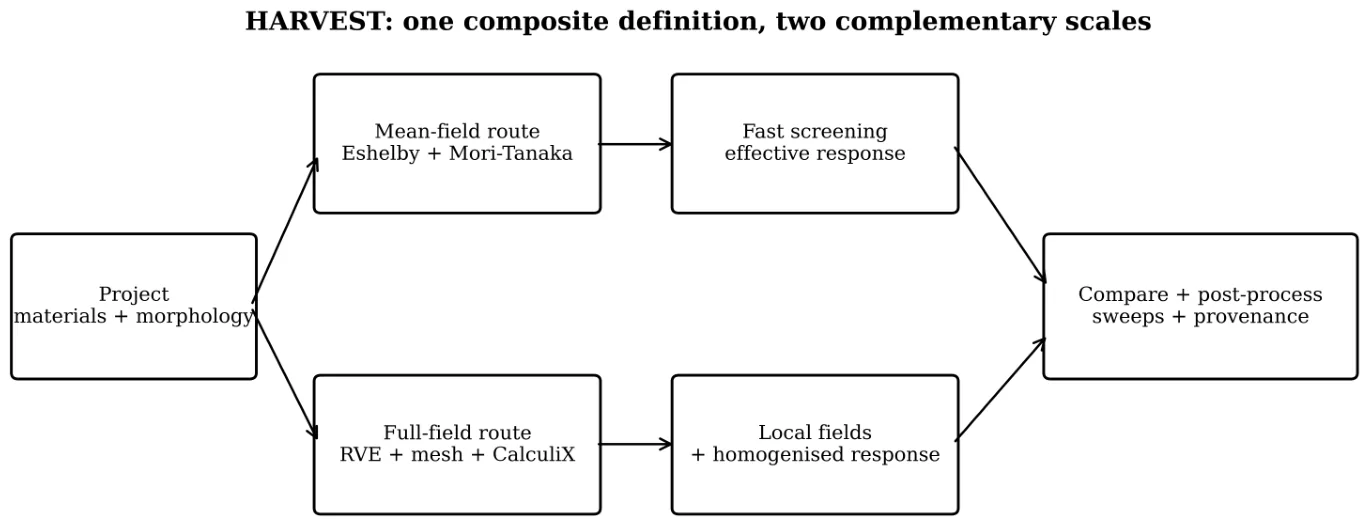
Scientific workflow represented by HARVEST. Mean-field and full-field analyses are complementary routes coordinated by common project, result and provenance concepts. The current development version retains documented compatibility adapters for established numerical modules.

HARVEST integrates mean-field homogenisation, RVE generation, FE model preparation and solution, homogenisation, parameter studies and post-processing. Figure 1 summarises the scientific workflow. Application services interpret a common project definition and select either the mean-field or full-field route. Established numerical implementations remain accessible through compatibility adapters while the package architecture is migrated incrementally.

Table 2 records the software version evaluated in this study.

The numerical formulation is material-agnostic. Constituent stiffness, phase fraction and morphology enter as data, allowing the same algorithms to represent ceramic-, metal- or void-containing composites. The examples and validation context in this paper focus on polymer matrices, which provide the intended scientific emphasis and the strongest available experimental datasets.

### 2.2. Mean-Field Route

For a two-phase composite with matrix stiffness $C_{m}$, inclusion stiffness $C_{i}$ and inclusion volume fraction *f*, the dilute concentration tensor is

(1) $$A^{\mathrm{dil}}={\left[I+S:C_{m}^{-1}:\left(C_{i}-C_{m}\right)\right]}^{-1}.$$

Here, $A^{\mathrm{dil}}$ is the fourth-order dilute strain-concentration tensor that relates the average inclusion strain to the remote matrix strain in the single-inclusion limit; I is the symmetric fourth-order identity tensor; S is the Eshelby tensor determined by the inclusion shape and the matrix Poisson ratio; and $C_{m}$ and $C_{i}$ are the fourth-order matrix and inclusion stiffness tensors. The quantity $C_{m}^{-1}$ is the matrix compliance, the colon denotes double tensor contraction, and the superscript −1 outside the square brackets denotes inversion of the resulting fourth-order operator. In the implementation, these tensors are represented by 6×6 Mandel matrices. The Mori–Tanaka concentration tensors follow [3]:

(2) $$A_{i}=A^{\mathrm{dil}}{\left[\left(1-f\right)I+fA^{\mathrm{dil}}\right]}^{-1},\;A_{m}=\frac{1}{1-f}\left(I-fA_{i}\right),$$

where $A_{i}$ and $A_{m}$ map the macroscopic strain to the phase-average inclusion and matrix strains, respectively; *f* is the inclusion volume fraction; and 1−f is the matrix volume fraction. The expressions assume 0≤f<1 and non-singular concentration operators. The effective stiffness is

(3) $$C^{\mathrm{eff}}=\left(1-f\right)C_{m}:A_{m}+fC_{i}:A_{i}.$$

Here, $C^{\mathrm{eff}}$ is the homogenised fourth-order stiffness tensor satisfying $\bar{\sigma }=C^{\mathrm{eff}}:\bar{\varepsilon }$. The two terms in Equation (3) are the matrix and inclusion contributions to the macroscopic stress, weighted by their phase volume fractions. All operations use Mandel notation [33], preserving the tensor inner product in the 6×6 representation. The implementation includes analytical Eshelby tensors for spheres, circular cylinders and spheroids, together with numerical quadrature for a general triaxial ellipsoid. In the local frame of a spheroid, the distinguished axis is axis 1 and

(4) $$\left(a_{1},a_{2},a_{3}\right)=\left(c,a,a\right),\;\alpha =\frac{a_{1}}{a_{2}}=\frac{c}{a},$$

where $a_{1}=c$ is the semi-axis along the distinguished local material direction, $a_{2}=a_{3}=a$ are the equal transverse semi-axes, and α is the dimensionless aspect ratio. Accordingly, α>1 denotes a prolate inclusion and α<1 an oblate inclusion. Stating the axis ordering is essential because the same scalar aspect ratio produces a different directional stiffness if assigned to another local axis.

The current production mean-field kernel evaluates aligned inclusions. For the verification and design study, an independent fourth-order rotation and quadrature layer averages the aligned tensor over prescribed orientation distributions. This layer uses the same Mandel convention and is separate from the supported application programming interface (API), providing a controlled assessment of the orientation operations.

Linear-elastic, generalised-Maxwell/Prony viscoelastic and J2 elastoplastic constituent classes share a uniform incremental interface. For nonlinear constituents, the matrix algorithmic tangent is isotropically projected for the Eshelby evaluation while the concentration step retains the current tangent, following the established tangent-homogenisation approach [34]. Only the linear-elastic branch is used for the quantitative studies reported here.

### 2.3. Full-Field Route and Microstructure Representation

**Figure 2:**
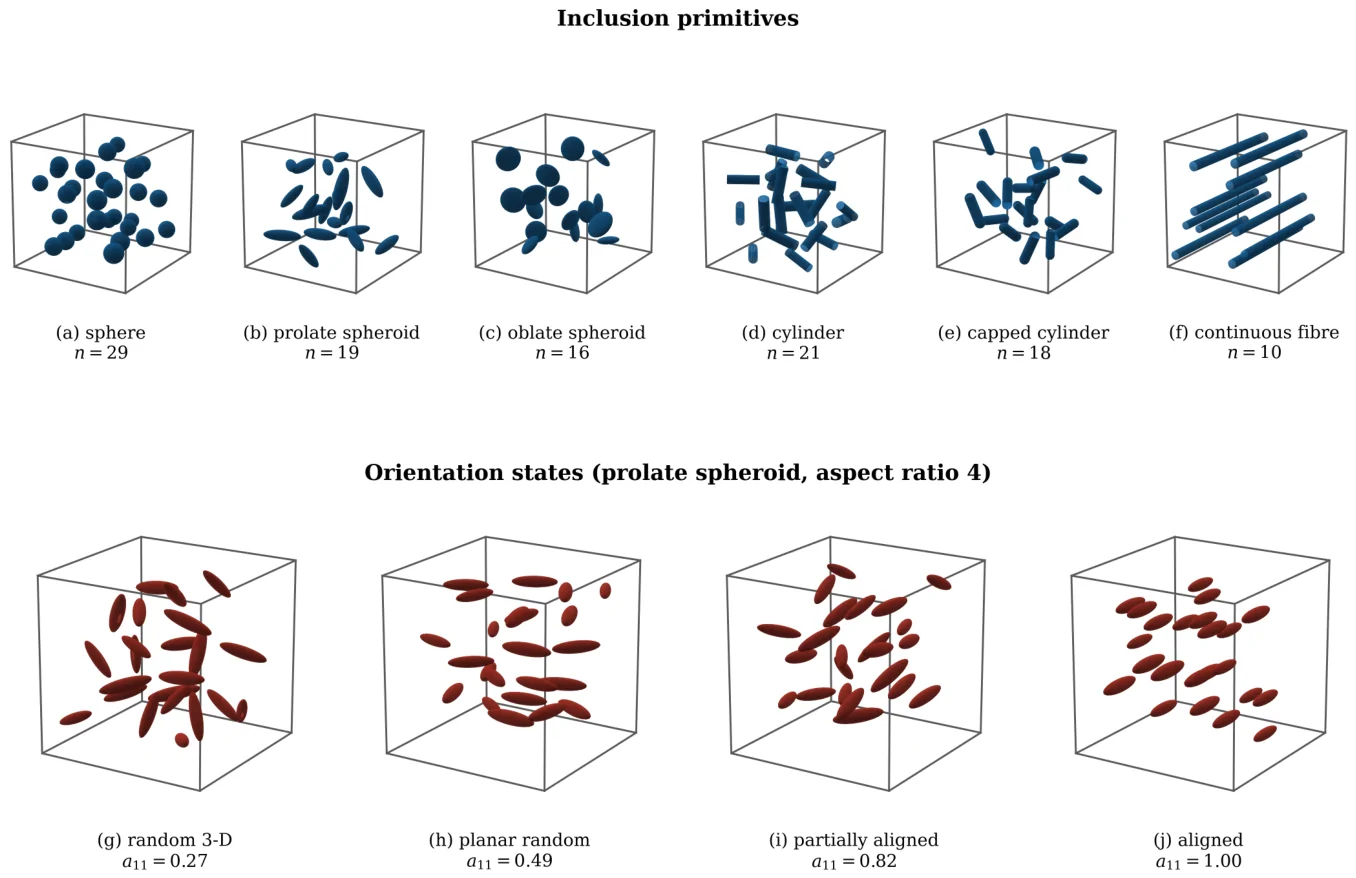
Illustrative HARVEST-compatible geometry records. (**a**–**f**) inclusion primitives used by the RVE workflow; *n* is the number of inclusions in each record. (**g**–**j**) finite-sample orientation states for prolate spheroids of aspect ratio 4. The measured $a_{11}$ values approach the theoretical random, planar and aligned limits as the sample size increases.

The full-field route describes inclusion families through material, morphology, fraction, size and placement settings. Current geometry support includes ellipsoids, hollow ellipsoidal shells, finite and capped cylinders, axis-aligned continuous fibres and interphase layers. Structured C3D8 discretisation provides a robust low-cost route based on element classification, while optional Gmsh [35] generation supports conformal C3D4 and C3D10 meshes. Figure 2 is an illustrative gallery generated by the deterministic geometry-record script using the same shape and orientation conventions. Orientation is summarised by the second-order tensor a=〈p⊗p〉, where p is the unit vector along the distinguished inclusion axis; $a_{11}=\langle p_{1}^{2}\rangle$ measures alignment with axis 1. The gallery supports visualisation and convention checks.

Published stochastic generators include algorithms for cylindrical and spherical fillers [36] and for spatially distributed discontinuous fibres [37]. They provide methodological context for specifying filler placement and orientation independently of the constituent properties.

**Figure 3:**
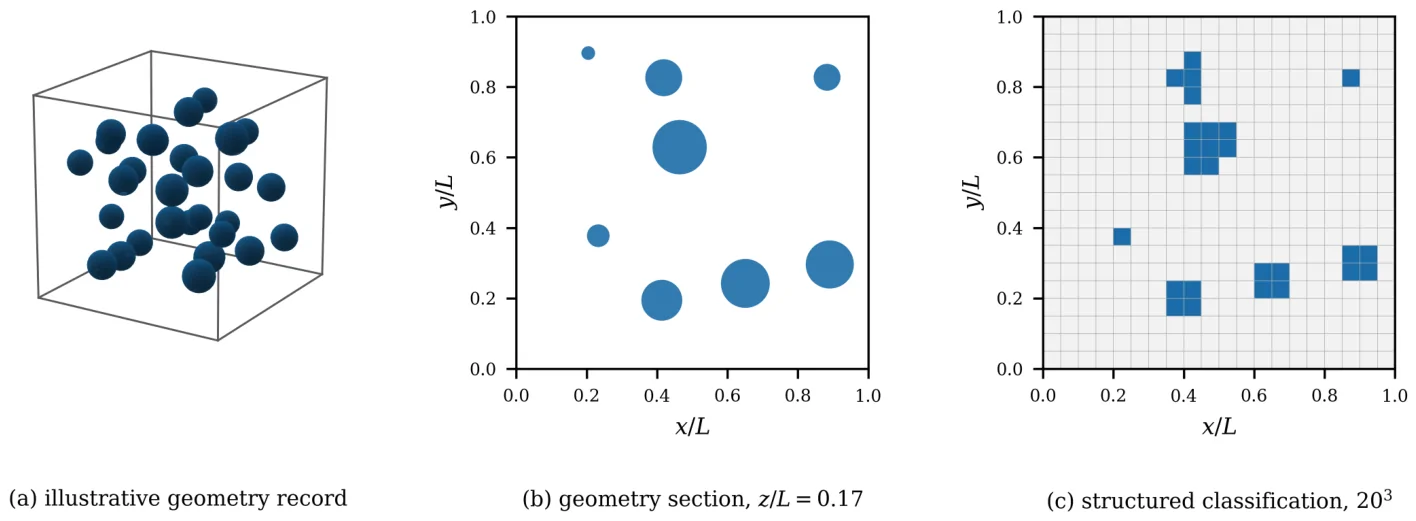
Illustration of geometry-to-discretisation transfer. (**a**) deterministic illustrative geometry record; (**b**) section at z/L=0.17; (**c**) centroid-based structured classification on a $20^{3}$ grid. Panel (**c**) illustrates the geometric bias of a voxel-like discretisation.

A deterministic seed controls stochastic generation, allowing a cell to be reconstructed. The geometry record stores centres, dimensions, orientation and family identity for downstream meshing and post-processing. The distinction between geometry and discretisation is visible in Figure 3: centroid classification on a $20^{3}$ structured grid introduces a stair-stepped interface and can omit a small cross-section at a selected plane. This behaviour reflects the geometric approximation introduced by centroid-based structured discretisation.

Sohn’s element-carving method combines periodic meshing with local refinement around inclusions [38]. Together with the voxel/tetrahedral comparison of Tüfekci et al. [13], this work motivates assessing interface resolution alongside the nominal mesh size.

### 2.4. RVE Boundary Conditions and Homogenisation

A periodic cell is decomposed into a macroscopic deformation and a periodic fluctuation,

(5) $$u\left(x\right)=\bar{\varepsilon }\;x+\tilde{u}\left(x\right).$$

In this decomposition, x is the material-point position, u(x) is its displacement, $\bar{\varepsilon }$ is the prescribed macroscopic small-strain tensor, and $\tilde{u}\left(x\right)$ is the periodic displacement fluctuation. The first term is the affine macroscopic displacement and the second carries the microstructural perturbation while taking equal values at corresponding points on opposite periodic faces. The present implementation sets the macroscopic rigid-body rotation to zero, so the affine displacement gradient is represented by the symmetric tensor $\bar{\varepsilon }$; the engineering shear satisfies ${\bar{\gamma }}_{12}=2{\bar{\varepsilon }}_{12}$. For matched points $x^{+}$ and $x^{-}$ on opposite faces, the periodic displacement constraint is

(6) $$u\left(x^{+}\right)-u\left(x^{-}\right)=\bar{\varepsilon }\;\left(x^{+}-x^{-}\right).$$

Here, $x^{+}$ and $x^{-}$ are corresponding points on opposite RVE faces; $x^{+}-x^{-}$ is the associated cell-lattice translation; and the right-hand side is the displacement jump generated by the prescribed macroscopic strain. Equation (6) enforces periodic fluctuations while allowing the cell to undergo the required average deformation. The current implementation uses deterministic spatial hashing and transverse-coordinate matching, followed by explicit face, edge and corner ownership. The procedure stops with an explicit error when opposite faces are nonconforming or pairing is ambiguous. Geometry determines the correspondence, while ownership prevents duplicated constraints at edges and corners.

Kinematic uniform boundary conditions (KUBC) prescribe the affine displacement on the full boundary and provide an upper bound for a finite RVE. Static uniform boundary conditions (SUBC), when available, provide a lower bound, while periodic boundary conditions generally produce an intermediate response for statistically representative cells. The archived quantitative campaign reported here uses KUBC, and the PBC machinery is assessed structurally through its constraint tests.

**Figure 4:**
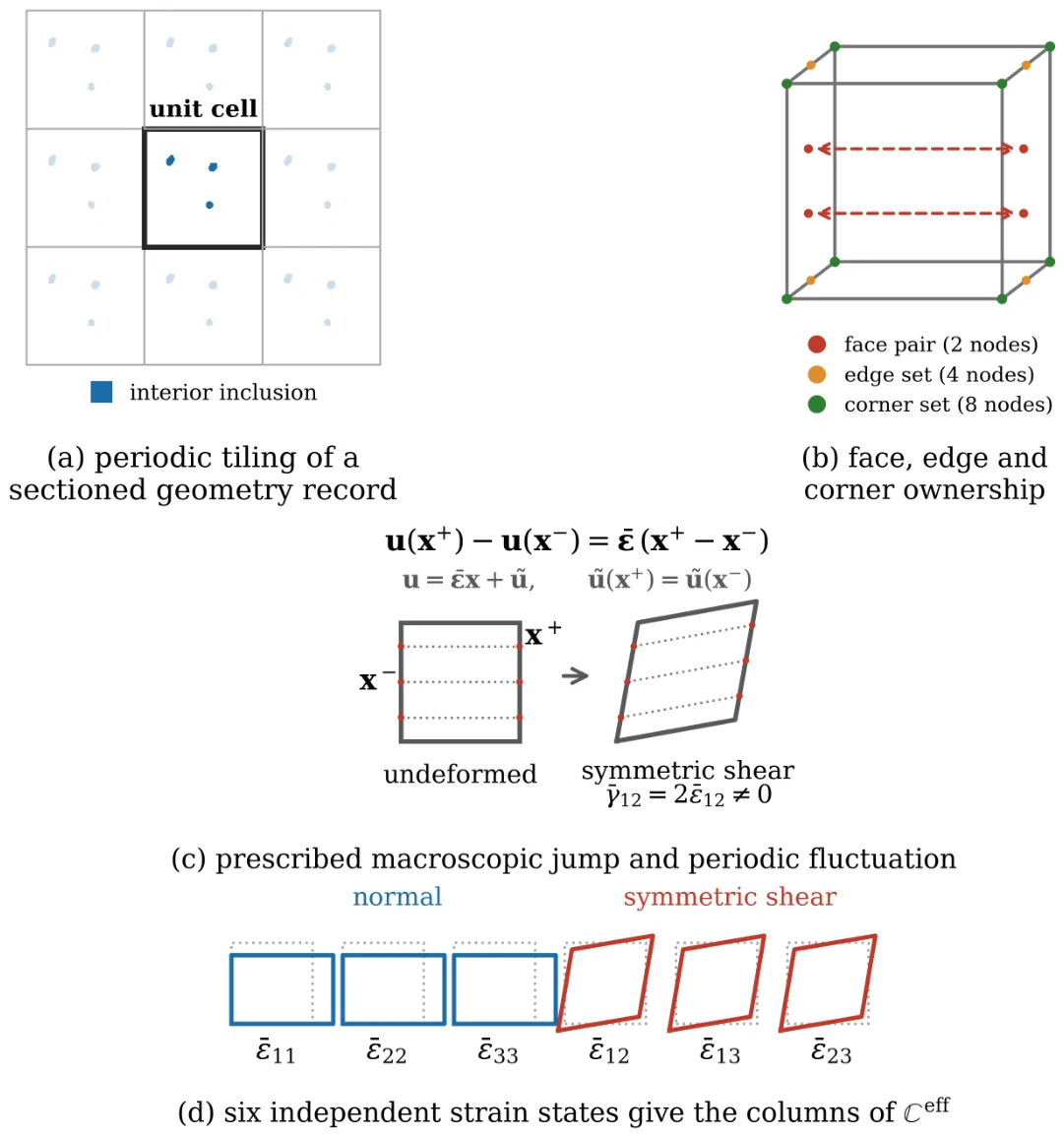
Periodic-cell construction. (**a**) periodic tiling of a sectioned geometry record; (**b**) face, edge and corner ownership used to avoid duplicate constraints; (**c**) displacement-jump condition in Equation (6), illustrated for symmetric small-strain shear with zero macroscopic rotation and ${\bar{\gamma }}_{12}=2{\bar{\varepsilon }}_{12}$; (**d**) six independent macroscopic strain states used to assemble $C^{\mathrm{eff}}$.

Six independent macroscopic strain states are solved to assemble the effective stiffness. For each load case,

(7) $$\bar{\sigma }=\frac{1}{V}\int_{V}\sigma \left(x\right)\;dV,\;C_{ij}^{\mathrm{eff}}={\bar{\sigma }}_{i}^{\left(j\right)}/{\bar{\varepsilon }}^{\left(j\right)},$$

where *V* is the RVE volume, σ(x) is the local Cauchy-stress vector, and $\bar{\sigma }$ is its volume average. The indices i,j∈{1,…,6} follow the selected Mandel ordering: *i* identifies the macroscopic stress component and *j* the independent load case. The scalar ${\bar{\varepsilon }}^{\left(j\right)}$ is the non-zero prescribed strain amplitude in load case *j*, so the second relation assembles column *j* of $C^{\mathrm{eff}}$; no summation over *j* is implied. Element-volume weighting is used in the post-processing. Figure 4 summarises the periodic construction and the six strain states.

### 2.5. Solver and Provenance Boundary

CalculiX [39] is external to the normal source and wheel distribution. Solver selection, capability checks, execution and result status are accessed through one validation and execution boundary. A missing executable, unsupported requested capability, malformed manifest, failed job or stale/incomplete output stops the analysis with an explicit failure status. Project, solver and result paths are separated through runtime and run-layout abstractions, reducing the risk that a post-processor reads a file from an earlier directory layout or a previous run.

The canonical harvest command exposes project, mean-field, RVE, solve, post-processing, sweep, diagnostics and graphical user interface (GUI) routes. PySide6 is the primary GUI framework, while parts of the established RVE workbench remain in an isolated compatibility process.

## 3. Verification Programme

### 3.1. Evidence Hierarchy

The verification programme prioritises independent analytical identities and limiting reductions, followed by independently written tensor operations and solver-neutral structural tests of periodic constraints. Cross-route comparisons are interpreted through known limits, bounds and ordering because mean-field and finite-cell KUBC calculations solve different idealised problems. Experimental comparisons form the subsequent physical-model validation.

### 3.2. Spherical Mean-Field Kernel

**Figure 5:**
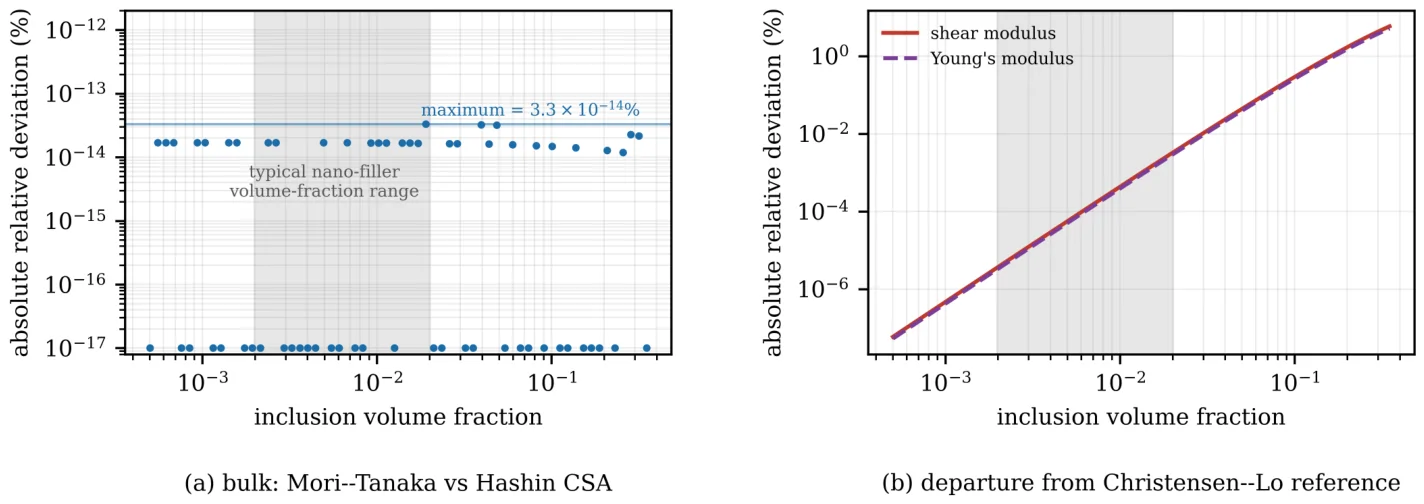
Independent mean-field verification for stiff spherical inclusions in an epoxy-like matrix. (**a**) Mori–Tanaka bulk modulus versus the exact Hashin CSA bulk branch; (**b**) shear and Young’s-modulus departure from the Christensen–Lo analytical composite-sphere reference. The shaded interval indicates 0.2–2 vol%, representative of many nanoparticle-modified polymers.

For isotropic spherical inclusions, the Mori–Tanaka bulk modulus coincides with the Hashin composite-sphere assemblage (CSA) result [40]. This equality is an exact property of the scheme and provides a sharp implementation test. Across the tested volume-fraction range, the maximum relative bulk difference is $3.3\times 10^{-16}$, as shown in Figure 5a.

The shear branch is compared with the independent analytical three-phase, generalised self-consistent reference of Christensen and Lo [41]. This three-phase analytical composite-sphere model provides an independent reference for the direction and magnitude of the Mori–Tanaka departure. Figure 5b shows a monotonic difference that remains small in the dilute polymer-nanocomposite range and grows as the inclusion fraction increases.

### 3.3. Layered-Sphere Reductions and Coated-Particle Approximation

The bulk branch of the Hervé–Zaoui layered-sphere solution [42] was implemented independently through radial transfer matrices. It is an exact solution of the concentric layered-sphere boundary-value problem in the branch tested. Four reductions were used to establish the reference implementation itself before it was used to assess HARVEST approximations. Table 3 reports the deviations.

The vanishing-shell deviation decreases from $3.8\times 10^{-4}$ at $t=10^{-3}$ to $3.8\times 10^{-10}$ at $t=10^{-9}$, consistent with the geometrically vanishing phase. The sequential coated-particle approximation used in the polymer design study was then compared with a separate two-stage analytical composite-sphere construction using the Hashin bulk branch and Christensen–Lo shear branch. The study combines four core-diameter/interphase-thickness pairs—(12 nm, 2 nm), (12 nm, 5 nm), (20 nm, 5 nm) and (80 nm, 5 nm)—with interphase-to-matrix modulus ratios of 0.5, 3 and 10 and core fractions of 0.5 and 2 vol%, giving 24 configurations. Across these cases, the largest absolute difference in Young’s modulus is 0.417%, and the median is 0.071%. The comparison quantifies the accuracy of the sequential approximation over the tested domain.

### 3.4. Orientation and Mandel-Convention Cross-Checks

The reproduction layer contains two independent routes to an isotropic tensor: direct averaging of a rotated fourth-order stiffness over a uniform orientation distribution and Frobenius-norm isotropic projection [34]. For a transversely isotropic input, the two agree to a relative difference of $1.624\times 10^{-14}$. Conversion from Mandel to fourth-order form and back has a maximum absolute round-trip error of $2.274\times 10^{-13}$ in the test scale, and a random rotation leaves an isotropic tensor invariant to $3.183\times 10^{-12}$. These checks are particularly valuable because a missing $\sqrt{2}$ Mandel shear factor can produce plausible normal moduli while corrupting shear and orientation averages.

### 3.5. Periodic-Constraint Tests

The periodic constraint generator is tested without invoking CalculiX. Opposite-face counts must match; ambiguous or missing pairs produce explicit errors; pair generation is deterministic under shuffled node order; face, edge and corner ownership must be unique; and an affine displacement field must satisfy every generated equation to machine precision for all six strain cases. The implementation also distinguishes full three-dimensional PBC from an in-plane xy option that deliberately leaves the *z* faces free. These tests establish the emitted kinematics and ownership logic for the implemented constraint generator.

### 3.6. Archived Full-Field Campaign Against Bounds

**Figure 6:**
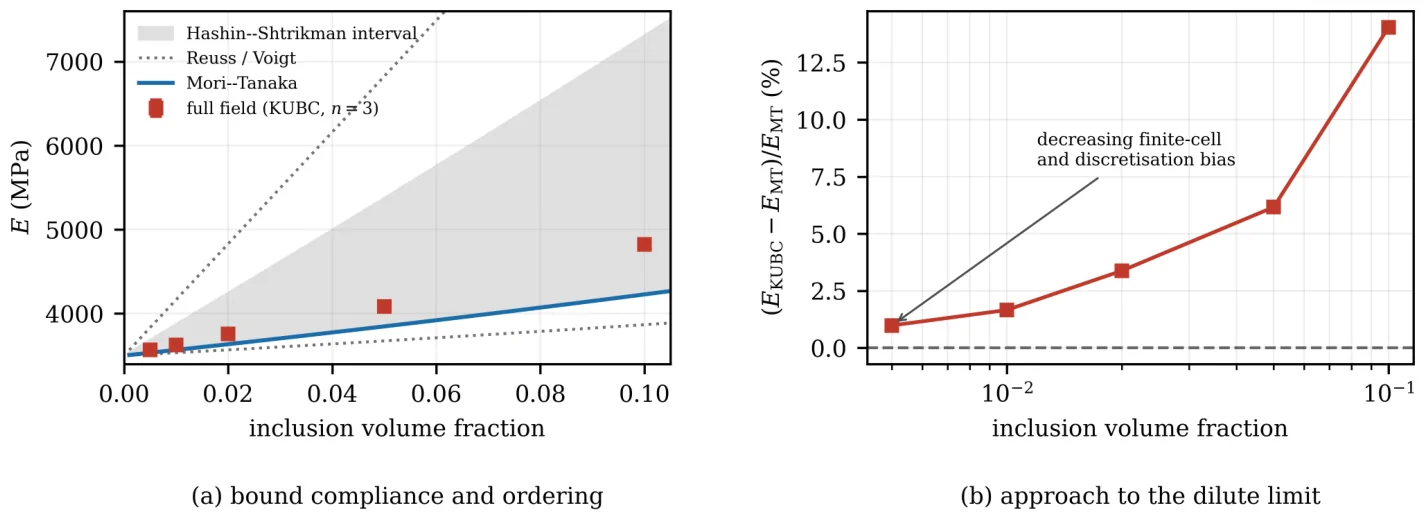
Archived mean-field/full-field comparison. (**a**) bound compliance and ordering for the KUBC campaign; error bars show three-realisation standard deviations. (**b**) relative KUBC excess over Mori–Tanaka as the dilute limit is approached. The figure is regenerated from the archived aggregate CSV.

An archived HARVEST/CalculiX epoxy/silica-type nanocomposite campaign provides end-to-end evidence for the full-field route. The matrix properties are $E_{m}=3500\mathrm{MPa}$ and $\nu_{m}=0.35$; spherical inclusions have $E_{i}=70\mathrm{GPa}$ and $\nu_{i}=0.20$. Five inclusion fractions were analysed using three stochastic realisations, $20^{3}$ structured C3D8 cells and KUBC. The Hashin–Shtrikman (HS) bounds [43] and the Mori–Tanaka (MT) estimate provide the comparison intervals in Table 4. Table 4 and Figure 6 summarise the archived aggregate results.

Every KUBC mean lies within the Hashin–Shtrikman interval and below the Voigt bound. The observed ordering is Reuss ≤ MT/HS^−^ ≤ KUBC ≤ HS^+^ ≤ Voigt for all five points. The KUBC–MT difference decreases from 14.0% at f=0.10 to 1.0% at f=0.005, while the largest realisation standard deviation is 0.38% of the mean. The excess over MT is consistent with the upper-bound character of KUBC and the over-stiffening tendency of a structured centroid discretisation. The campaign is used to assess bound compliance and realisation scatter; mesh-convergence evidence is outside its scope.

### 3.7. Scope of the Verification Evidence

The evidence above establishes numerical identities, controlled approximations, constraint behaviour and bound compliance for the cases tested. Physical accuracy remains governed by the constituent data and the assumptions of each micromechanical model. Phenomena such as cavitation, interface debonding, damage, fracture, fatigue and manufacturing defects require dedicated formulations and validation. Section 4 therefore evaluates effective-modulus predictions against published polymer-composite experiments and discusses the resulting model-form limitations.

## 4. Validation and Design Studies with Polymer-Based Nanocomposites

### 4.1. Validation Against Published Epoxy/HNT and Epoxy/CTBN Systems

The primary application uses the epoxy nanocomposites reported by Pir et al. [16]. The epoxy matrix was represented by the measured flexural modulus $E_{m}=2410.8\mathrm{MPa}$, Poisson’s ratio $\nu_{m}=0.35$ and density $\rho_{m}=1.15\;g\;cm^{-3}$; halloysite nanotube (HNT) was represented as a prolate elastic inclusion with E=140GPa, ν=0.25, density 2.53 g ${\mathrm{cm}}^{-3}$ and aspect ratio 30; carboxyl-terminated butadiene–acrylonitrile (CTBN) rubber was represented as a spherical soft inclusion with E=200MPa, ν=0.49 and density 0.98 g ${\mathrm{cm}}^{-3}$. The source study investigated 0.5 and 1 wt% HNT but 5 and 10 wt% CTBN; the two series represent distinct stiffening and toughening strategies rather than dosage-matched comparisons. HNT is a high-aspect-ratio stiff nanofiller that is effective at low content but sensitive to dispersion and agglomeration, whereas a compliant rubber phase is normally introduced at several weight per cent to activate rubber-domain toughening mechanisms [44,45,46,47]. Mass fractions were converted once to volume fractions using

(8) $$f=\frac{w/\rho_{i}}{w/\rho_{i}+\left(1-w\right)/\rho_{m}}.$$

Here, *w* is the inclusion mass fraction, $\rho_{i}$ and $\rho_{m}$ are the inclusion and matrix mass densities in consistent units, and *f* is the resulting inclusion volume fraction. The relation follows a two-phase mass balance and assumes no additional porosity or third phase. Table 5 reports the selected idealisation, measured flexural modulus and prediction. The experimental means and sample standard deviations are taken from the quasi-static three-point-bending results in Tables 6 and 7 of Pir et al. [16]. The comparison provides physical-model validation at the effective-modulus level using flexural data; specimen-scale processing and the assumed constituent properties remain part of the comparison.

The HNT predictions are close to the measured values only under an aligned directional interpretation. The independently computed three-dimensional random-orientation average gives 2455.4 and 2500.3 MPa for the two HNT fractions, respectively. The aligned and random assumptions therefore differ by 6.9 and 13.6% even though the constituent data and mass fraction are identical. The large sensitivity shows that morphology and measurement direction must be recorded. Casting, moulding and flexural specimen preparation can induce preferential alignment, but a measured orientation tensor would be required to remove this modelling ambiguity.

The CTBN cases underpredict by 7.03 and 2.25%. A two-phase linear-elastic model represents elastic softening while omitting cavitation, debonding, shear-band formation and the strain-rate dependence associated with rubber-toughened thermosets. Huang and Kinloch [48] used FE analysis to examine local stresses and plastic shear-band development in rubber-modified epoxies. Hsieh et al. [49] identified shear yielding and particle debonding followed by plastic void growth in silica-modified epoxies and examined hybrid rubber/silica systems. These mechanisms define additional physics required to extend stiffness screening to toughness prediction; the present modulus differences also depend on the assumed constituent properties and morphology.

The different HNT and CTBN contents in the source study reflect their distinct reinforcement mechanisms rather than a universal dosage ratio. Relative to the unmodified flexural modulus, the source data show increases of 9.86 and 17.26% at 0.5 and 1 wt% HNT, but decreases of 1.12 and 13.58% at 5 and 10 wt% CTBN. Independent HNT studies likewise report stiffening at low loadings and demonstrate that the optimum depends on dispersion, matrix chemistry and agglomeration; maxima around 3 wt% were reported for the particular epoxy systems examined by Ravichandran et al. [44] and Srivastava and Pandey [45]. Ye et al. [50] reported improved impact performance in HNT-filled epoxy while retaining flexural properties, illustrating why stiffness and toughening criteria can favour different formulations. Independent CTBN studies identify a stiffness–toughness trade-off whose magnitude depends on cross-link density, rubber-domain morphology, cavitation and matrix shear yielding [46,47]. These comparisons support the direction of the HARVEST predictions while explaining why one elastic idealisation cannot reproduce every measured magnitude or fracture mechanism.

**Figure 7:**
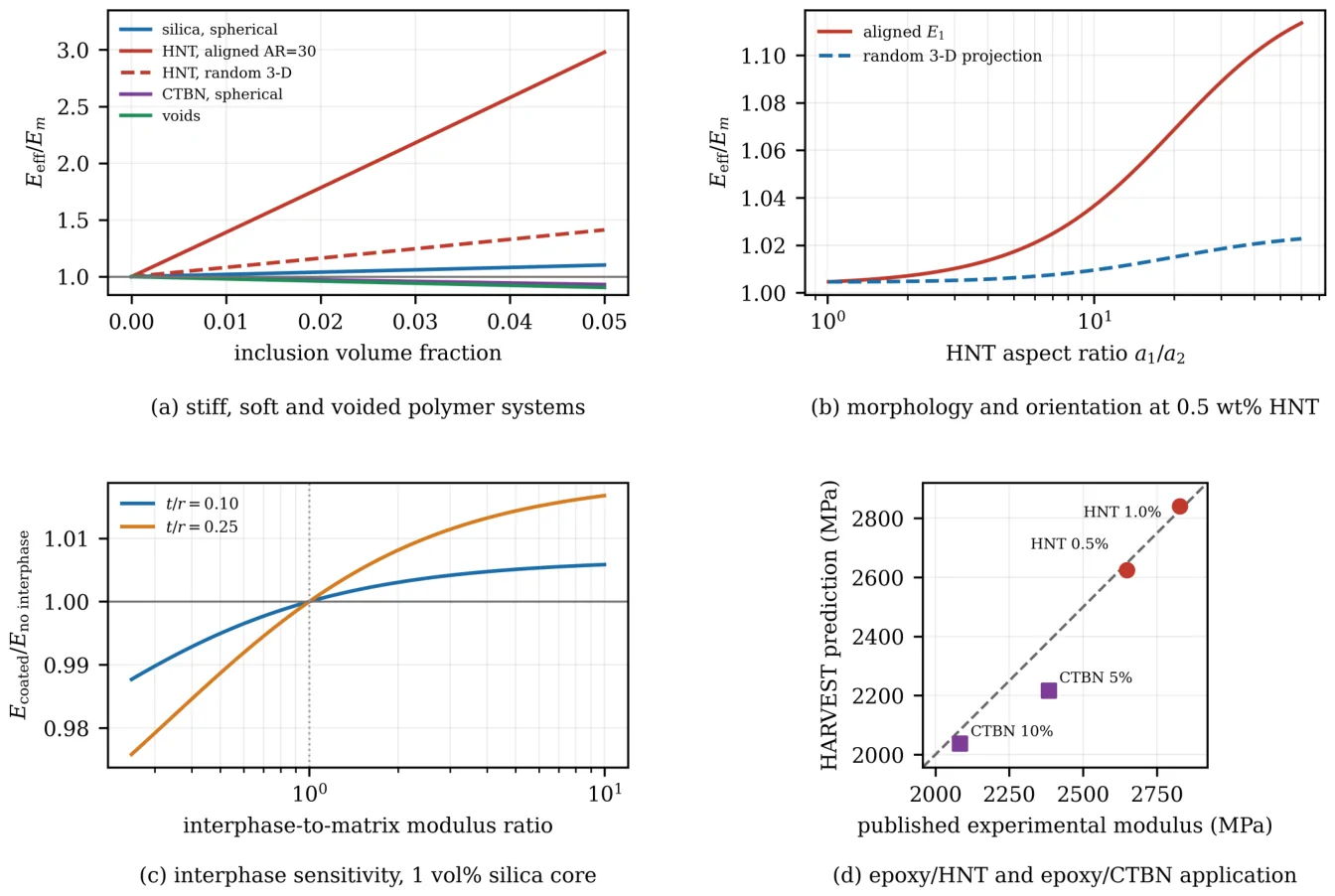
Polymer-focused design study. (**a**) stiffness contrast and morphology at 0–5 vol%; (**b**) HNT aspect ratio and orientation at 0.5 wt%; (**c**) sensitivity to a compliant or stiff interphase around a 1 vol% silica core; (**d**) selected predictions against the experimental quasi-static flexural moduli reported in Tables 6 and 7 of Pir et al. [16].

Additional specimens at intermediate or common mass fractions would extend material-specific experimental validation. The common-volume numerical sweep instead isolates stiffness contrast and morphology while controlling for density. Figure 7a performs the latter comparison over 0–5 vol% as a numerical design study.

### 4.2. Polymer Micromechanics Design Map

Figure 7 extends the four experimental points into a controlled parameter study. Figure 7a uses the same epoxy matrix to compare spherical silica, aligned and randomly oriented HNT, spherical CTBN and voids. At 5 vol%, the predicted normalised modulus is 1.10 for silica, 2.98 for aligned HNT, 1.41 for randomly oriented HNT, 0.93 for CTBN and 0.90 for voids. The comparison shows that “reinforcement fraction” alone is not a useful design coordinate without stiffness contrast and morphology.

Figure 7b isolates HNT shape and orientation at 0.5 wt%. Increasing the aspect ratio from approximately 1 to 60 raises the aligned prediction from 1.005 to 1.114 times the matrix modulus, whereas the random projection rises only to 1.023. The high aspect ratio has little macroscopic effect when its direction is averaged away at this low volume fraction. Figure 7c considers a 1 vol% silica core with interphase thickness ratios t/r=0.10 and 0.25. A compliant interphase reduces the predicted modulus, a stiff interphase raises it, and the larger shell amplifies both effects. Figure 7d places the published HNT and CTBN points against the selected predictions from Table 5.

### 4.3. Extension to Polymer Membranes

Mixed-matrix membrane studies provide an important extension of the same modelling problem. PAN reinforced with silica or HNT [17,18], PES containing CNF [19], and PVDF- or PSf/PVP-based membranes containing CNC and CNF [20,21] all combine a polymer matrix, a high-aspect-ratio or particulate phase and substantial morphology changes introduced by processing. Mean-field analysis is useful for screening the constituent contrast, but pore topology, agglomeration and local interaction may require an explicit cell. These studies illustrate relevant application classes. The quantitative polymer validation reported here uses the epoxy/HNT and epoxy/CTBN systems, while the membrane datasets provide broader application context.

## 5. Discussion

### 5.1. Generality of the HARVEST Framework and Scope of the Polymer Validation

The numerical kernels act on phase tensors, fractions, geometry and solver-neutral result concepts, allowing the same framework to represent multiple material classes. This study focuses validation on epoxy-based nanocomposites and discusses interphases, rubber modification, voids, high-aspect-ratio fillers and polymer membranes. The polymer emphasis defines the validation domain, while the software architecture remains general to composite micromechanics.

Beyond polymers, Tüfekci [51] combined mean-field and RVE-based homogenisation of porous Ti–6Al–4V with static and prestressed modal analysis of fan blades. This earlier application illustrates the transfer of effective material properties to structural analysis; polymer nanocomposites remain the experimental validation basis of the present study.

### 5.2. When the Two Routes Add Value

Mean-field analysis is most useful for rapid sensitivity studies and screening. Figure 7 can be generated without an external FE solver and immediately shows whether stiffness contrast, orientation or interphase assumptions dominate a polymer formulation. Full-field analysis becomes valuable when local fields, spatial interaction, pore topology or nonlinear localisation are the quantities of interest. Maintaining both routes within a common project and provenance framework allows selected designs to progress from screening to detailed analysis using unchanged constituent definitions.

The two routes solve different idealised problems at finite volume fraction. A finite KUBC cell is an upper-bound boundary-value problem, whereas Mori–Tanaka is a mean-field estimate with its own morphology assumptions. Their relationship is assessed through limits, bounds and ordering.

Gentieu et al. [52] examined the dependence of analytical predictions on particle shape and concentration using periodic full-field models of highly filled particulate composites. Their two-dimensional study provides a complementary basis for separating morphology-related differences from the boundary-condition and realisation effects considered here.

### 5.3. Applications, Practical Advantages and Extension Beyond the Demonstrated Cases

The current HARVEST framework supports applications beyond the four epoxy formulations used for validation. Table 6 identifies representative polymer-composite decisions that can be addressed with the existing routes. In each case, the mean-field model supports broad screening, while the full-field model is reserved for selected designs requiring local fields or explicit topology.

The principal practical advantages are the preservation of one constituent and morphology definition across the two scales, deterministic reconstruction of stochastic studies, automated parameter sweeps, explicit solver/result status and reproducible post-processing. These features reduce manual transfer errors and allow a large design space to be screened cheaply before computational effort is concentrated on a smaller set of full-field candidates. Convergence studies and experimental calibration remain necessary.

For a new case that uses an existing inclusion geometry and the current linear-elastic, Prony-series viscoelastic or J2 elastoplastic constituents, expansion mainly requires new project data and an independent validation dataset rather than changes to the numerical kernel. A genuinely new constitutive response requires a state-update and consistent tangent implementation; a new morphology requires geometry generation, meshing and phase-labelling support; a new FE programme requires a solver backend, deck writer and result parser; and a new physical claim requires analytical, numerical or experimental validation appropriate to that claim. Each extension therefore combines software implementation, regression testing and physical validation.

Measured-morphology modelling provides a further extension. Guven and Cinar [53] reconstructed particle-filled epoxy RVEs from micro-computed-tomography images and compared voxel- and geometry-based representations. For scale-dependent stress analysis, Tüfekci et al. [54] applied integral nonlocal post-processing to conventional FE results for multi-phase materials. These extensions would require image reconstruction and phase labelling, or kernel and length-scale inputs, respectively, together with independent verification of the added modelling route.

### 5.4. Current Limitations

Current material limitations include general anisotropic nonlinear behaviour, hyperelasticity, cyclic plasticity, fatigue, progressive damage, cohesive debonding and coupled thermal/electrical physics. Full-field gaps include nonconforming or mortar PBC, measured or image-derived microstructures, mature woven and braided representations and a published end-to-end Hill–Mandel validation dossier. A general production orientation-distribution workflow is also less mature than the aligned Eshelby kernel.

The quantitative FE evidence in this paper covers spherical inclusions, structured cells, KUBC and three realisations. It establishes an end-to-end path, theoretical ordering and realisation scatter for this configuration; high-order homogenisation accuracy requires additional mesh and formulation studies.

The software architecture is being migrated incrementally. Canonical command-line interface (CLI) and service boundaries are established, while selected mature GUI and numerical routes retain compatibility wrappers or isolated processes.

## 6. Conclusions

This study introduces HARVEST as a coordinated platform for mean-field and full-field composite micromechanics. Its principal contribution is a traceable workflow in which constituent properties, morphology, loading conventions and result definitions are shared across rapid mean-field screening and selective full-field RVE analysis. This cross-route consistency reduces manual transfer errors and allows differences between the two routes to be interpreted through their mechanics, morphology assumptions and boundary conditions.

Independent verification reproduced the Hashin composite-sphere-assemblage bulk branch to machine precision, cross-validated the orientation operations to a relative difference of $1.6\times 10^{-14}$, and gave a largest absolute Young’s-modulus difference of 0.417% for the sequential coated-particle approximation across 24 cases. The archived full-field campaign with kinematic uniform boundary conditions remained within the Hashin–Shtrikman interval at five volume fractions and showed less than 0.4% realisation scatter. These results establish implementation consistency for the reported kernels and the expected theoretical ordering for the tested full-field configuration.

Validation with polymer-based nanocomposites captured the opposite stiffness trends produced by halloysite-nanotube and carboxyl-terminated butadiene–acrylonitrile-rubber additions. The selected halloysite-nanotube predictions differed from the published flexural moduli by less than 1%, whereas the rubber cases showed larger differences consistent with the omission of cavitation, debonding and damage mechanisms from the elastic model. The parametric studies further showed that aspect ratio, orientation and interphase properties can influence the predicted polymer response as strongly as filler fraction. The current development version can support screening and local-field studies for particulate, tubular, rubber-modified, porous, syntactic and mixed-matrix polymer systems. HARVEST remains under active development as a general material simulation tool oriented towards composite materials; extensions to new constitutive laws, morphologies or solvers require corresponding software verification and physical validation.

## Figures and Tables

**Table 1:** Functional comparison with selected composite-micromechanics tools based on the cited publications and official documentation.

Tool	Published Scope	Relationship to HARVEST
HARVEST	Mean-field homogenisation, synthetic RVE generation, CalculiX-based full-field analysis, parameter studies and post-processing. The development platform is Python-based; an external CalculiX installation is required for FE solution.	Coordinates both routes through one project and provenance layer. Its present constitutive, process-modelling and validation coverage remains narrower than that of mature commercial platforms.
Digimat [28]	Broad nonlinear multiscale material and structure modelling, including process–material–part links for plastics, composites, metals and elastomers.	Provides broader industrial material and process coverage; HARVEST instead emphasises a coordinated mean-field/full-field research workflow with traceable inputs and result provenance.
Ansys Material Designer [29]	Workbench module for FE-based RVE generation, analysis and transfer of homogenised properties to later structural analyses.	Provides a strongly integrated full-field workflow; HARVEST adds a paired mean-field/full-field research workflow and explicit result provenance.
EasyPBC [11]	Abaqus plug-in that automates periodic constraints and effective elastic properties for user-created cuboid RVEs.	Focuses on full-field homogenisation for user-created RVEs; mean-field homogenisation and microstructure generation remain separate workflows.
MicroStructPy [30]	Python package for statistical two- and three-dimensional microstructure generation, verification and meshing.	Provides complementary geometry and meshing capability; a separate solver and homogenisation workflow are required.

**Table 2:** Software snapshot and implemented capability scope.

Item	Recorded State
Development version	0.7.0.dev0
Implementation language	Python 3.10 or later; paper-specific calculations tested with NumPy 2.3.5, SciPy 1.17.0 and Matplotlib 3.10.8
Canonical user interface	harvest command with project, mean-field, RVE, solve, post-processing, sweep, diagnostics and graphical user interface (GUI) routes
Mean-field capability	Eshelby/Mori–Tanaka elastic response; incremental linear viscoelastic and J2 constituent models; analytical inclusion shapes
Full-field capability	Synthetic 3-D RVEs, structured C3D8 and optional Gmsh C3D4/C3D10 meshes, kinematic uniform boundary conditions (KUBC) and matched-mesh periodic boundary conditions (PBC), CalculiX decks and result post-processing
Verification baseline	More than 600 automated unit, invariant, parser, constitutive, homogenisation, periodic-boundary-condition, solver and workflow checks
Development status	Development version; platform testing and application-specific validation are ongoing

**Table 3:** Limiting reductions of the independently implemented layered-sphere bulk reference.

Reduction	Relative Deviation
Single shell → two-phase Hashin CSA, f=0.001–0.4	≤$1.7\times 10^{-16}$
Vanishing shell → two-phase result, $t=10^{-9}$	$3.8\times 10^{-10}$
Shell properties equal to matrix	$1.6\times 10^{-16}$
All phases identical → homogeneous medium	$1.7\times 10^{-16}$

**Table 4:** Archived full-field campaign. Moduli are in MPa; full-field values are mean ± sample standard deviation for three KUBC realisations.

*f*	Reuss	HS^−^ = MT	Full Field (KUBC)	HS^+^	Voigt
0.005	3516.7	3533.3	3568.1±3.9	3685.6	3832.5
0.010	3533.6	3566.9	3626.2±1.7	3871.6	4165.0
0.020	3567.8	3635.1	3757.9±8.7	4244.6	4830.0
0.050	3674.5	3847.1	4084.3±6.0	5374.9	6825.0
0.100	3867.4	4227.9	4821.6±18.3	7308.8	10,150.0

**Table 5:** Polymer application results. Experimental quasi-static three-point-bending moduli are taken from Tables 6 and 7 of Pir et al. [16]; the reported uncertainty is the sample standard deviation. HNT is represented by an aligned prolate inclusion of aspect ratio 30, whereas CTBN is represented by spherical soft inclusions.

System	*f* (vol%)	$E_{\exp}$ (MPa)	$E_{\mathrm{pred}}$ (MPa)	Error (%)
HNT 0.5 wt%	0.228	2648.6±54.3	2624.9	−0.89
HNT 1.0 wt%	0.457	2827.0±102.4	2840.5	+0.48
CTBN 5 wt%	5.817	2383.8±124.6	2216.3	−7.03
CTBN 10 wt%	11.535	2083.5±61.5	2036.5	−2.25

**Table 6:** Representative polymer-composite applications of the current framework.

Material Class or Application	Design Question	Appropriate HARVEST Use
Particle- or nanotube-modified thermosets	How do filler content, stiffness contrast, aspect ratio, orientation and interphase properties change effective stiffness?	Mean-field sweeps for screening; selected RVEs for localisation and inclusion-interaction assessment
Rubber-toughened polymers	What elastic softening accompanies the added compliant phase, and where do local hydrostatic and shear stresses develop?	Elastic screening and full-field stress analysis; predictive toughness requires additional cavitation, debonding and damage models
Mixed-matrix and nanocomposite membranes	How do reinforcing particles, pores and processing-induced morphology alter stiffness and local stress?	Mean-field constituent screening followed by explicit RVE analysis when pore topology or agglomeration is important
Porous and syntactic polymer systems	How do void fraction, hollow-particle wall thickness and coating/interphase stiffness affect density and effective response?	Coated-particle mean-field studies and full-field verification of local shell/matrix stress concentrations
Short-fibre or platelet composites	Which aspect ratio and orientation state provide the required anisotropic stiffness?	Directional mean-field studies and statistically sampled RVEs, subject to the present orientation and geometry limits

## Data Availability

The original contributions presented in this study are included in the article. Further inquiries can be directed to the author.
